# Supplementary material for: Human Pancreatic Islets React to Glucolipotoxicity by Secreting Pyruvate and Citrate
Source: Nutrients. 2023 Nov 15;15(22):4791. doi: 10.3390/nu15224791 (PMC10674605; doi:10.3390/nu15224791)
Supplement: Supplementary file 1 [file nutrients-15-04791-s001.zip › FigS1_revised.pdf]

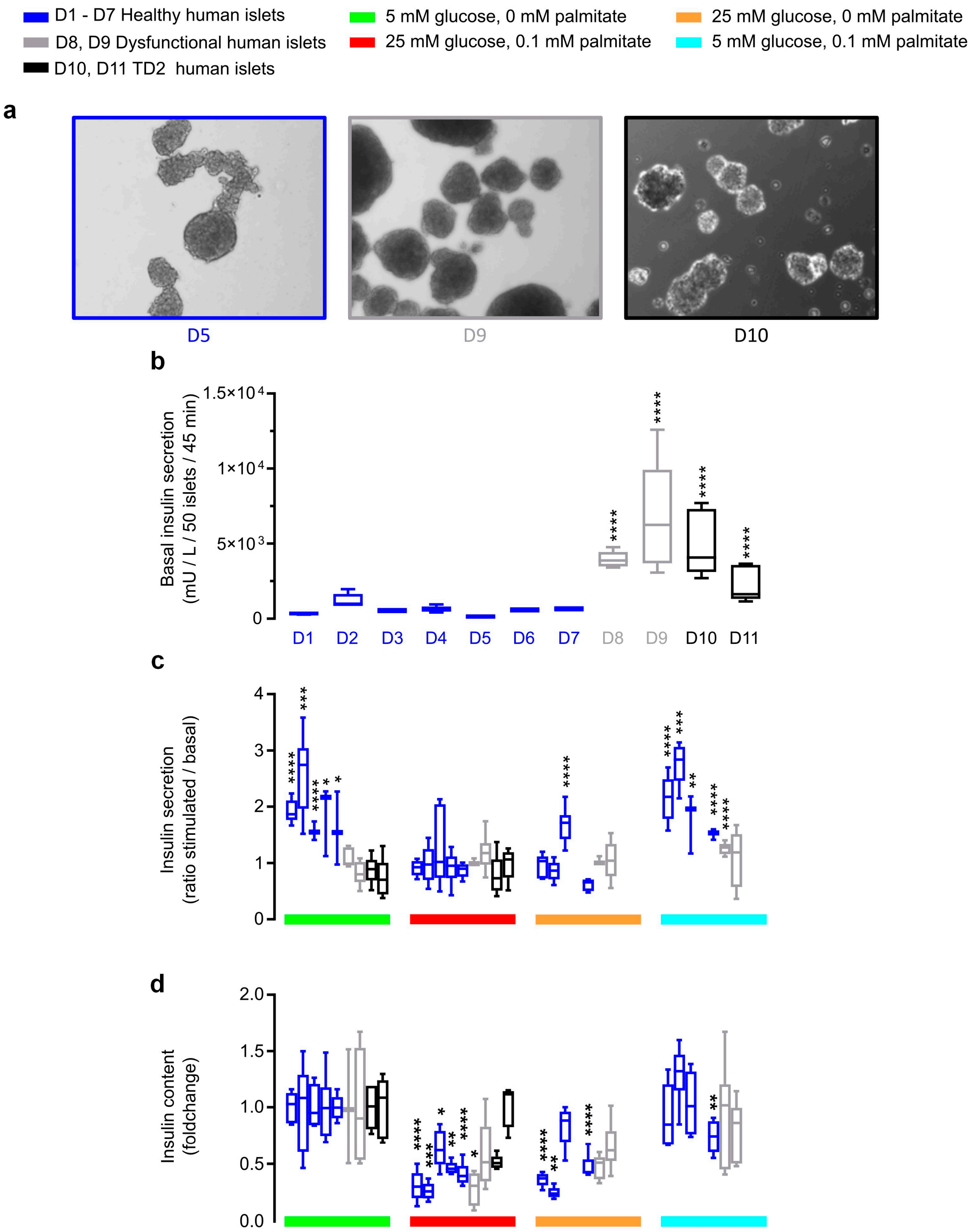

Figure S1, related to Figures 1A and 1B: Individual islet batch behavior regarding insulin secretion response after glucolipotoxicity. (a) Phase image of representative isolated human islets from donor D5, D9 and D10 in culture. (b)

Boxplots showing quantification of insulin secretion during the first 45 minutes at 5.5 mM of glucose after 48 hours of culture in control medium (D1 to D9 are non-diabetic donors, D10 and D11 are type 2 diabetic donors). ****$p<0.0001$ to compare dysfunctional and TD2 islets with mean of healthy islets. (c, d) Boxplots showing individual Stimulated/Basal insulin secretion (c) and corresponding cellular insulin content (IC) (d) of human pancreatic islets during GSIS after 48 h of culture with indicated glucose and palmitate concentration (n= 6 insulin quantification per batch of islets). A student t-test was conducted to compare the level of secreted insulin after basal (5.5 mM) and stimulated (25 mM) glucose addition in the medium *$p <0.05$, **$p<0.01$, ***$p<0.001$, ****$p<0.0001$.
