# Supplementary material for: Human Pancreatic Islets React to Glucolipotoxicity by Secreting Pyruvate and Citrate
Source: Nutrients. 2023 Nov 15;15(22):4791. doi: 10.3390/nu15224791 (PMC10674605; doi:10.3390/nu15224791)
Supplement: Supplementary file 1 [file nutrients-15-04791-s001.zip › FigS3_revised.pdf]

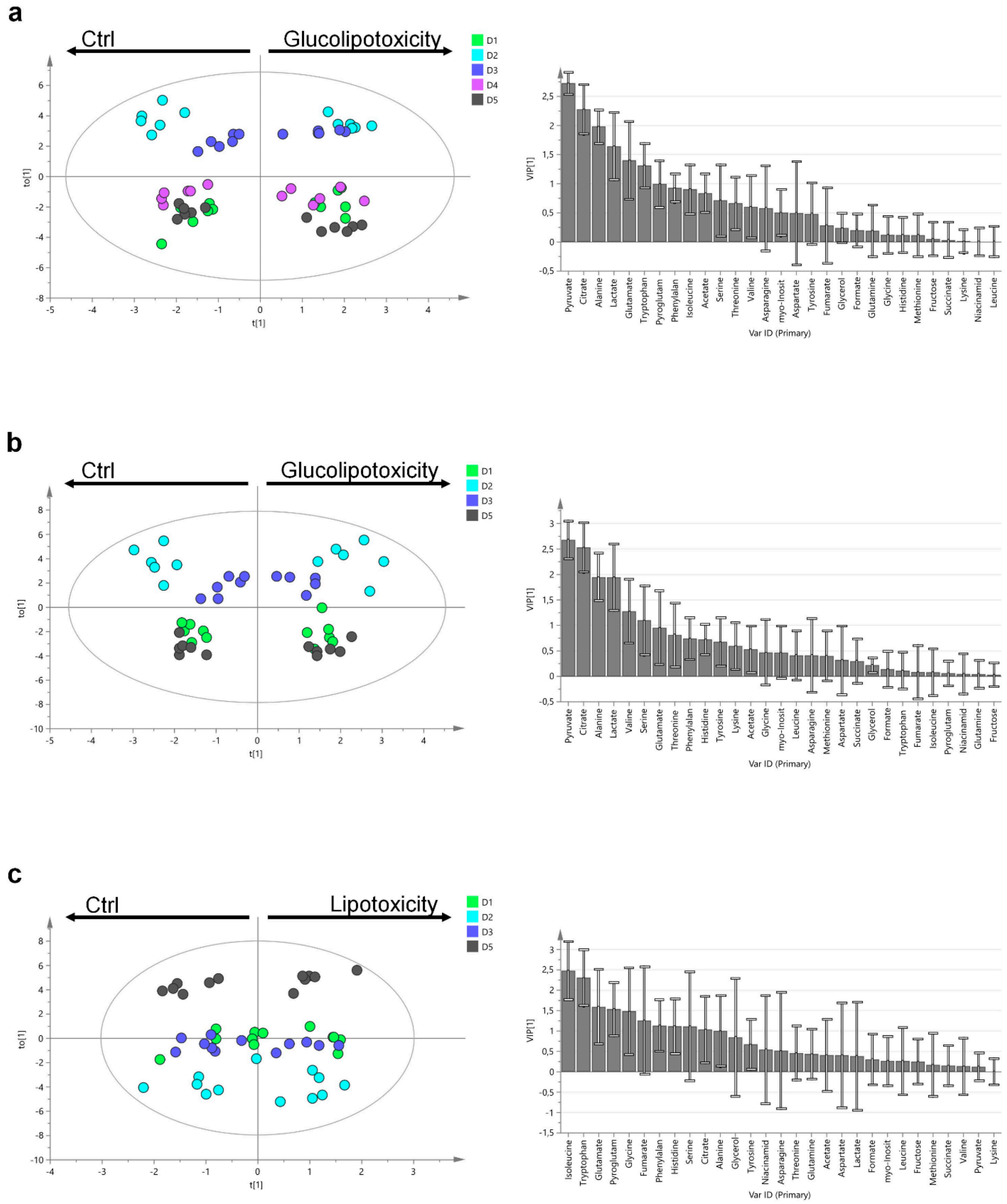

a
Ctrl
Glucolipotoxicity
D1
D2
D3
D4
D5
to[1]
t[1]
VIP[1]
Var ID (Primary)
Pyruvate
Citrate
Alanine
Lactate
Glutamate
Tryptophan
Pyroglutam
Phenylalan
Isoleucine
Acetate
Serine
Threonine
Valine
Asparagine
myo-Inosit
Aspartate
Tyrosine
Fumarate
Glycerol
Formate
Glutamine
Glycine
Histidine
Methionine
Fructose
Succinate
Lysine
Niacinamid
Leucine
b
Ctrl
Glucolipotoxicity
D1
D2
D3
D5
to[1]
t[1]
VIP[1]
Var ID (Primary)
Pyruvate
Citrate
Alanine
Lactate
Valine
Serine
Glutamate
Threonine
Phenylalan
Histidine
Tyrosine
Lysine
Acetate
Glycine
myo-Inosit
Leucine
Asparagine
Methionine
Aspartate
Succinate
Glycerol
Formate
Tryptophan
Fumarate
Isoleucine
Pyroglutam
Niacinamid
Glutamine
Fructose
c
Ctrl
Lipotoxicity
D1
D2
D3
D5
to[1]
t[1]
VIP[1]
Var ID (Primary)
Isoleucine
Tryptophan
Glutamate
Pyroglutam
Glycine
Fumarate
Phenylalan
Histidine
Serine
Citrate
Alanine
Glycerol
Tyrosine
Niacinamid
Asparagine
Threonine
Glutamine
Acetate
Aspartate
Lactate
Formate
myo-Inosit
Leucine
Fructose
Methionine
Succinate
Valine
Pyruvate
Lysine

**Figure S3: Multivariate analyses to compare metabolic signature of healthy functional islets under control versus glucolipotoxicity (a), glucotoxicity (b), lipotoxicity (c).** (a-c) Partial least squares-discriminant analysis (PLS-DA) scores plot of data from 1 H NMR metabolomics analysis of islets after 48 h in control versus glucolipotoxicity (a), glucotoxicity (b) or lipotoxicity (c) condition. They all show the separation achieved according to treatment ((a) p-value= $9.25x10^{-21}$, ((b) p-value= $9.25x10^{-21}$, ((c) p-value= $9.25x10^{-21}$,). Colours correspond to different donors as show on the panel. The 3-class model is based on 1 predictive component and 2 orthogonal components ((a) N = 60, R2(X) = 0.512, R2(Y) = 0.898, and Q2 = 0.869, (b) N = 47, R2(X) = 0.632, R2(Y) = 0.877, and Q2 = 0.846, (c) N = 48, R2(X) = 0.599, R2(Y) = 0.767, and Q2 = 0.53,) and corresponding variables importance for the projection (VIP) that summarizes the importance of each variables on the PLS-DA model. The VIP ranking priority was according to the VIP values. Metabolites with VIP >1 are empirically considered has having a significant impact on the model discrimination power. All ellipses showed in the models represent the Hoteling T2 with 95% confidence. Each data point represents one islet supernatant sample, and the distance between points in the plot indicates the similarity between samples.
