# Supplementary material for: Human Pancreatic Islets React to Glucolipotoxicity by Secreting Pyruvate and Citrate
Source: Nutrients. 2023 Nov 15;15(22):4791. doi: 10.3390/nu15224791 (PMC10674605; doi:10.3390/nu15224791)
Supplement: Supplementary file 1 [file nutrients-15-04791-s001.zip › FigS4_revised.pdf]

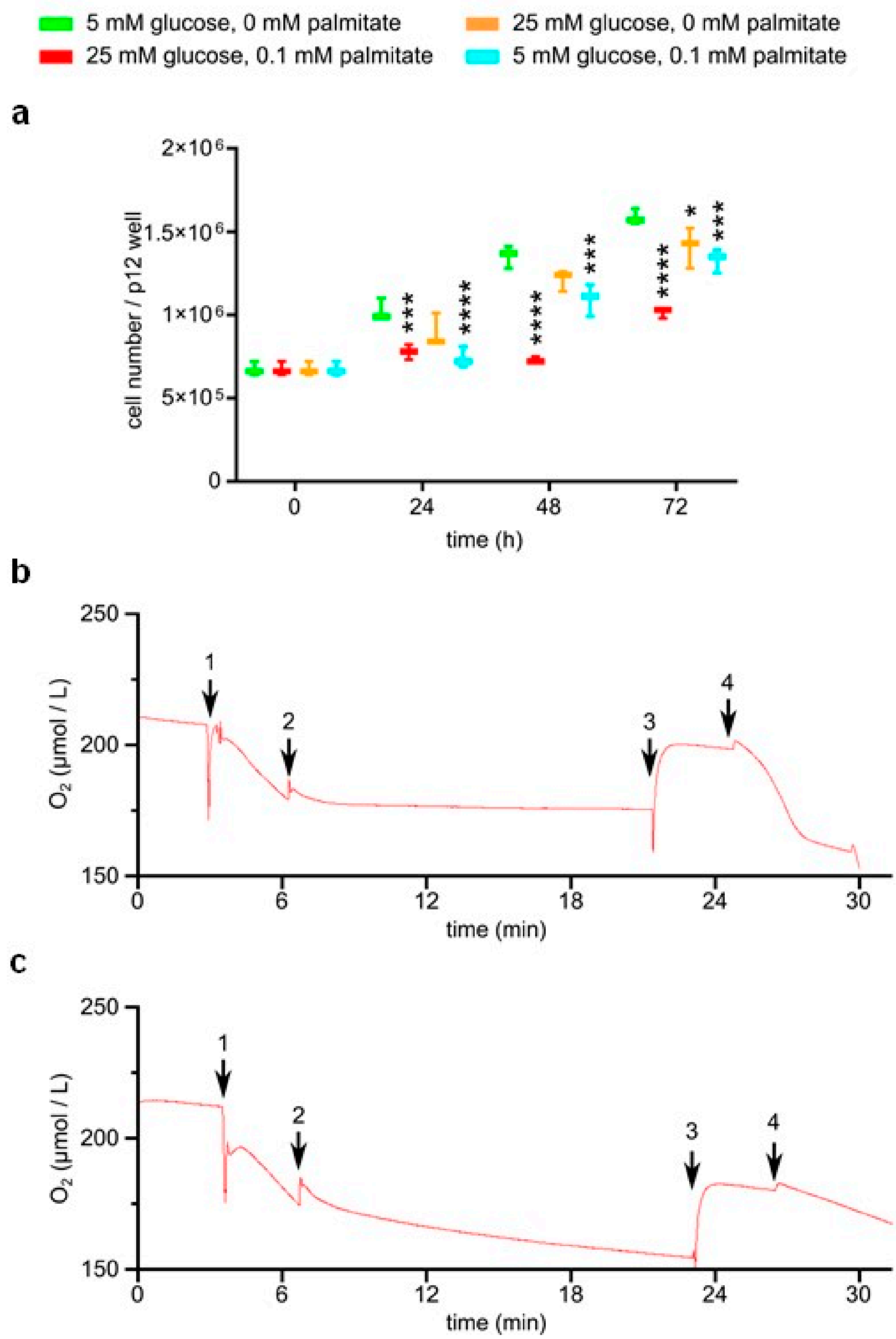

Fig. S4: INS-1E cell viability and permeabilized INS-1E cell oxygen consumption. (a) Cumulative cell survival as a function of time in control, gluco- lipo- and glucolipotoxicity condition concentrations obtained by trypan blue cell counting assay. (b, c) Representative traces of mitochondrial respiration in permeabilized INS-1E cells after 48 h in control (a) and glucolipotoxic (b) mediums. Compounds were added as indicated: 1: cells, 2: digitonin, 3: pyruvate, 4: ADP. * $p < 0.05$, *** $p < 0.001$, **** $p < 0.0001$.
