# Supplementary material for: Human Pancreatic Islets React to Glucolipotoxicity by Secreting Pyruvate and Citrate
Source: Nutrients. 2023 Nov 15;15(22):4791. doi: 10.3390/nu15224791 (PMC10674605; doi:10.3390/nu15224791)
Supplement: Supplementary file 1 [file nutrients-15-04791-s001.zip › FigS5_revised.pdf]

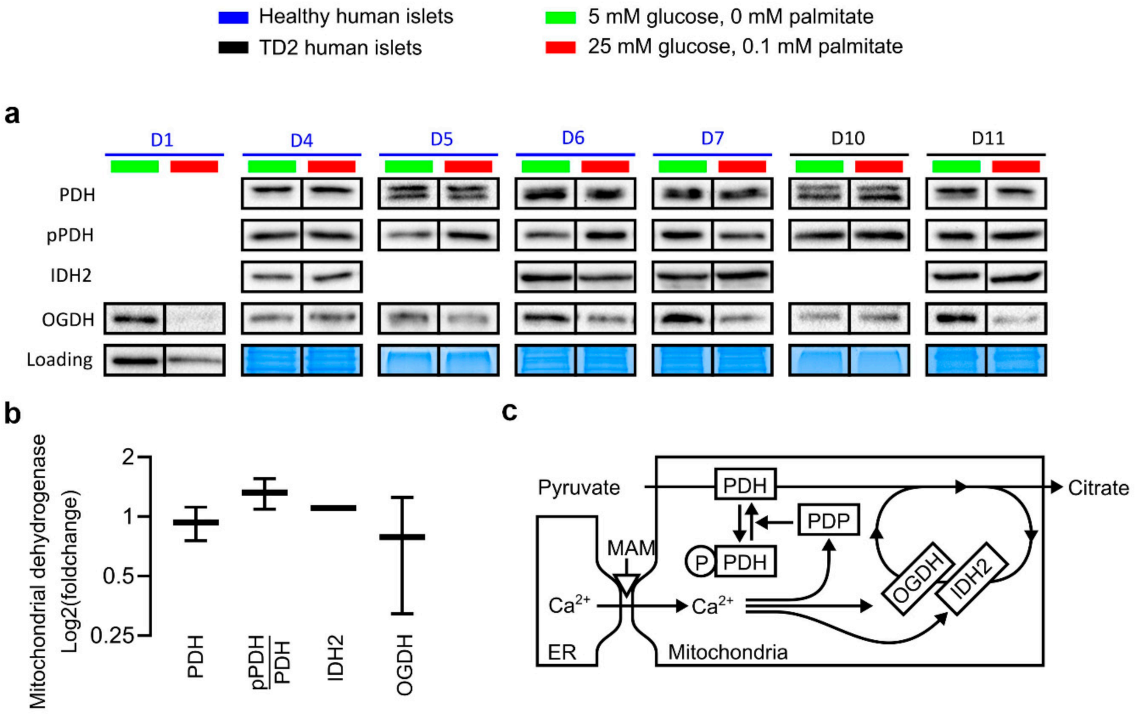

Figure S5, related to Figure 1i: Mitochondrial dehydrogenase expression and mitochondrial-endoplasmic reticulum contact quantification. (a) Representative Western blot related to Fig. 1i. (b) Quantitative analyses of PDH, pPDH, IDH2, OGDH in human pancreatic islets of TD2 islets (D10-D11) cultured 48 h under control or glucolipotoxicity. (c) A proposed model to link mitochondria- endoplasmic reticulum interaction (MAM), mitochondrial dehydrogenase and mitochondrial metabolite secretome related to this work and our previous results[22].
