# Supplementary material for: Human Pancreatic Islets React to Glucolipotoxicity by Secreting Pyruvate and Citrate
Source: Nutrients. 2023 Nov 15;15(22):4791. doi: 10.3390/nu15224791 (PMC10674605; doi:10.3390/nu15224791)
Supplement: Supplementary file 1 [file nutrients-15-04791-s001.zip › FigS6_revised.pdf]

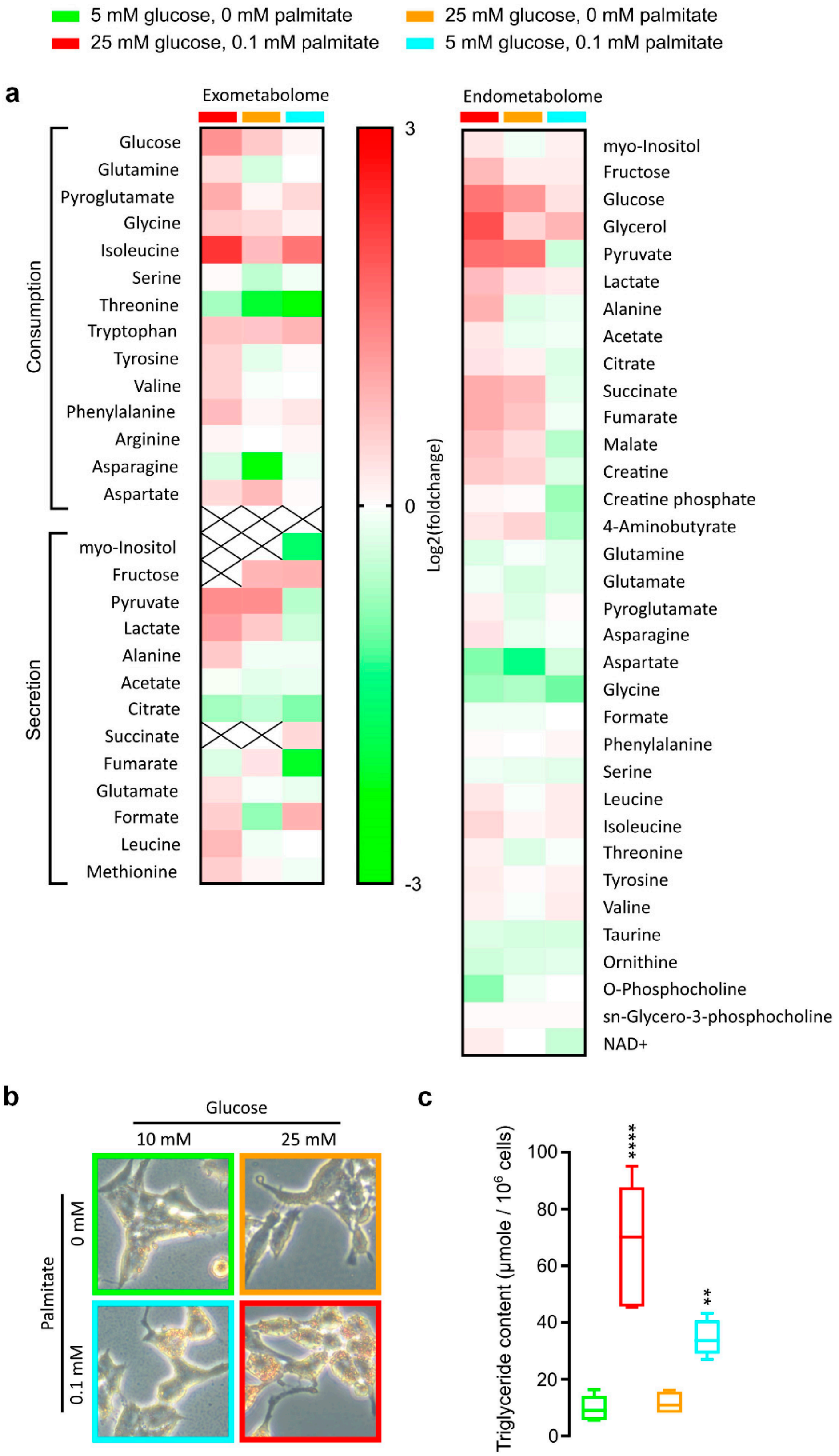

5 mM glucose, 0 mM palmitate
25 mM glucose, 0 mM palmitate
25 mM glucose, 0.1 mM palmitate
5 mM glucose, 0.1 mM palmitate
a
Exometabolome
Consumption
Glucose
Glutamine
Pyroglutamate
Glycine
Isoleucine
Serine
Threonine
Tryptophan
Tyrosine
Valine
Phenylalanine
Arginine
Asparagine
Aspartate
Secretion
myo-Inositol
Fructose
Pyruvate
Lactate
Alanine
Acetate
Citrate
Succinate
Fumarate
Glutamate
Formate
Leucine
Methionine
3
0
-3
Log2(foldchange)
Endometabolome
myo-Inositol
Fructose
Glucose
Glycerol
Pyruvate
Lactate
Alanine
Acetate
Citrate
Succinate
Fumarate
Malate
Creatine
Creatine phosphate
4-Aminobutyrate
Glutamine
Glutamate
Pyroglutamate
Asparagine
Aspartate
Glycine
Formate
Phenylalanine
Serine
Leucine
Isoleucine
Threonine
Tyrosine
Valine
Taurine
Ornithine
O-Phosphocholine
sn-Glycero-3-phosphocholine
NAD+
b
Glucose
10 mM
25 mM
Palmitate
0 mM
0.1 mM
c
Triglyceride content (µmole / 10^6 cells)
100
80
60
40
20
0
****
**

Figure S6, related to Figure 2: INS-1E exometabolome and endometabolome profiling and comparison between gluco-, lipo- and glucolipotoxicity. (a) Log2 Fold change of extracellular (left) and intracellular (right) metabolite concentrations of INS-1E cells after 48 h in corresponding glucose and palmitate conditions compared to control one. (b) Representative bright field images of INS-1E cells after 48 in corresponding toxicity. Lipid droplets were stained with oil red O solution. (c) Boxplots showing intracellular triglyceride concentration normalized by cell number after 48 h incubation with indicated culture conditions **(n=6 replicates per condition)**. Error bars represent standard deviation. **p<0.01, ****p<0.0001
