# Supplementary figures and images for: Human Pancreatic Islets React to Glucolipotoxicity by Secreting Pyruvate and Citrate
Source: Nutrients. 2023 Nov 15;15(22):4791. doi: 10.3390/nu15224791 (PMC10674605; doi:10.3390/nu15224791)

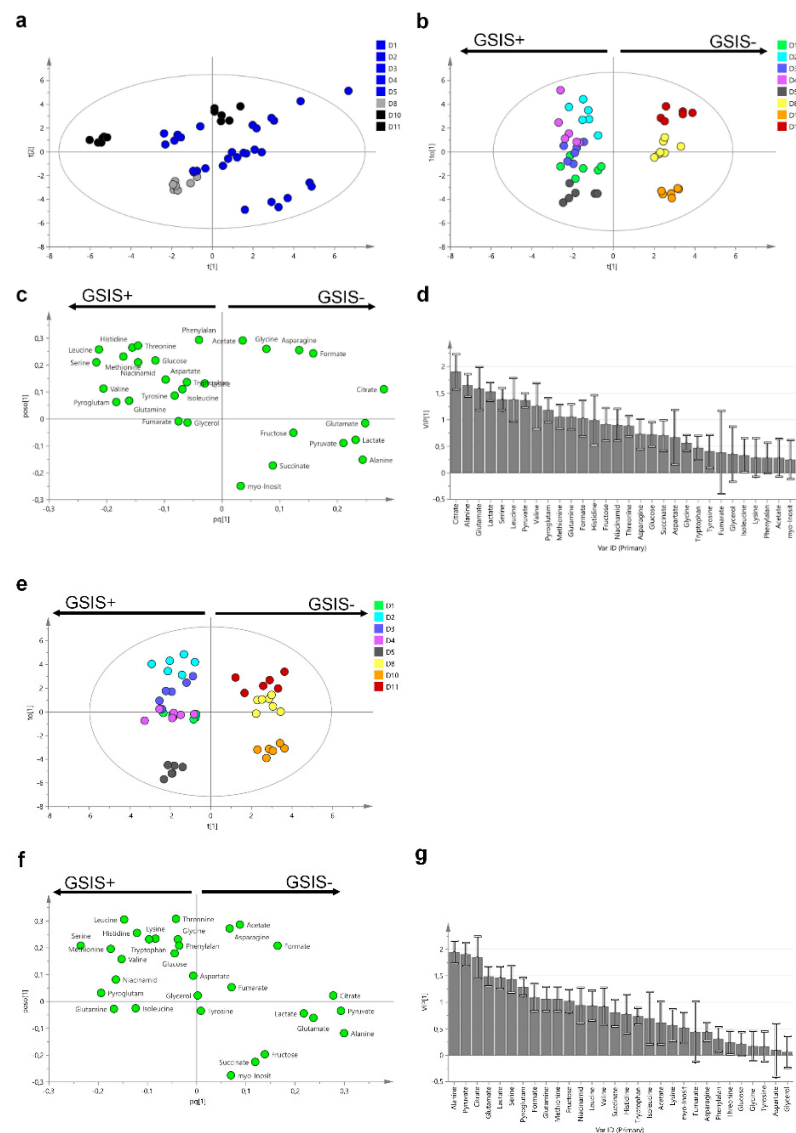

Supplement: Supplementary file 1 [file nutrients-15-04791-s001.zip › FigS2_revised.pdf]
